# Within-host selection of drug resistance in a mouse model of repeated interrupted treatment of *Plasmodium yoelii* infection

**DOI:** 10.1186/s12936-017-1860-6

**Published:** 2017-05-23

**Authors:** Suci Nuralitha, Josephine E. Siregar, Din Syafruddin, Andy I. M. Hoepelman, Sangkot Marzuki

**Affiliations:** 1 Eijkman Institute for Molecular Biology, Jakarta, Indonesia; 2 Internal Medicine and Infectious Diseases, University Medical Centre, Utrecht, Netherlands

**Keywords:** Within-host selection of atovaquone resistance, Mouse malaria model, *Plasmodium yoelii*, Repeated interrupted treatment

## Abstract

**Background:**

To study within-host selection of resistant parasites, an important factor in the development of resistance to anti-malarial drugs, a mouse model of repeated interrupted malaria treatment (RIT) has been developed. The characteristics of within host selection of resistance to atovaquone and pyrimethamine in *Plasmodium yoelii* was examined in such a model.

**Methods:**

Treatment of *P. yoelii* infected mice, with atovaquone or pyrimethamine, was started at parasitaemia level of 3–5%, interrupted when reduced to less than 0.4%, and restarted following parasitaemia recovery to the initial level. Treatment cycles were repeated until stable phenotype resistance was observed.

**Results:**

*Plasmodium yoelii* rapidly developed resistance to atovaquone (2.75 ± 1.06 cycles) and to pyrimethamine (5.4 ± 0.89 cycles) under RIT. A dose dependent phenomenon in the selection of atovaquone resistance mutations was observed. All mutations that underlie resistance to therapeutic doses of 0.3–1.44 mg kg^−1^ BW were found to be in the Qo2 domain of the cytochrome *b* gene (I258M, F267I/L/S, L271V, K272R, L271V and K272R). Those associated with lower doses of 0.01–0.03 mg kg^−1^ BW were in the Qo1 domain (M133I and T139S). The resistance mutations occurred at four of the 16 atovaquone putative drug binding sites suggested in *P. falciparum.*

**Conclusions:**

RIT of *P. yoelii* infected mice led to rapid development of resistance to atovaquone and pyrimethamine. The dose dependent selection of resistance mutants to atovaquone observed during RIT might reflect the outcome of two different causes of malaria treatment failure in human, repeated incomplete treatment with therapeutic dose and repeated inadequate treatment associated with sub-therapeutic dose, and need to be systematically investigated.

## Background

Within-host selection of resistant parasites, which precedes their spread in the population, is an important factor in the development of resistance to anti-malarial drugs, but still poorly understood. Animal models of malaria infection allow pharmacological manipulations in vivo. A model, based on cycles of repeated incomplete treatment of *Plasmodium berghei* infected mice, has been recently developed to study within host selection of resistant parasites to therapeutic doses of atovaquone and pyrimethamine [1]. Atovaquone is an inhibitor of mitochondrial respiratory complex III, and resistance to this anti-malarial drug is associated with genetic lesions in the mitochondrial cytochrome *b* (*cytb*) gene [2–9]. Pyrimethamine’s mechanism of action as inhibitor of *dihydrofolate reductase* (*dhfr*) is also well understood; resistance to this drug is associated with genetic lesions in the nuclear *dhfr* gene [10, 11].

Stable resistance to the anti-malarial atovaquone was found to be established within 2.5 treatment cycles [1]. For pyrimethamine, stable resistance developed after 5.5 cycles of treatment, consistent with the general observation that the rate of mutation in the mitochondrial DNA (mtDNA) is significantly higher than that of the nuclear DNA [12].

The above findings have major implications to the understanding of the development of anti-malarial drug resistance in human population. In early field trial of atovaquone, irrespective of the duration of therapy, a marked decrease in susceptibility to the anti-malarial was observed in the recrudescent parasites after the first treatment [13]. Resistance developed rapidly, in approximately 1 year, from the first introduction of pyrimethamine to population in 1967 [14]. It is thus crucial to confirm that the rapid development of resistance following repeated incomplete treatment is not an anomaly peculiar to *P. berghei*. Further, it is important to expand the study to examine the development of resistance over a wider range of atovaquone doses, as resistance resulting from repeated incomplete treatment with therapeutic dose in *P. berghei* is associated with mutations mostly in the quinone binding 2 (Qo2) domain of the *cytb* gene (Y268C/N/S, L271V and K272R) [1]; in contrast, isolation of atovaquone resistant mutants of *P. berghei* and *Plasmodium yoelii* by serial technique with increasing doses of the drug (ST) [4, 5], resulted in resistant parasites with underlying mutations in or near the quinone binding 1 (Qo1) domain (M133I, L144S, V284F).

In the present study, the characteristics of within host selection of resistance to atovaquone and pyrimethamine during repeated interrupted treatment (RIT) of *P. yoelii* infected mice have been examined, employing atovaquone doses ranging from approx. 10 to 1500 times ED_50_. *Plasmodium berghei* and *P. yoelii* were the first murine models of malaria developed to test drug efficacy [15, 16]. As for *P. berghei,* stable atovaquone resistance was already established after 2.5 treatment cycles. At the higher doses of more than 100 times ED_50_, the resistance was found to be associated with mutations in the Qo2 domain. At 30 times ED_50_ or less, however, the underlying resistance mutations were found to be in the Qo1 domain. The authors suggest that the experimental conditions associated with the above results mimic two different causes of malaria treatment failure—repeated incomplete treatment with therapeutic dose as in the RIcT mouse model [1], and repeated inadequate treatment associated with sub-therapeutic dose.

## Methods

### Mice, malaria parasites and drugs

Specific pathogen free BALB/c mice were obtained from the Animal Resources Centre, Murdoch, Western Australia and maintained in pathogen free animal house facility of the Eijkman Institute. *Plasmodium yoelii nigeriensis* was obtained from Toyama Medical and Pharmaceutical University, Japan. The *P. yoelii* stocks were screened for several pathogens, and maintained by passages in mice as previously described [1].

Atovaquone was kindly provided by Dr. Mary Pudney of the Wellcome Research Laboratories, UK. Pyrimethamine was purchased from Sigma-Aldrich Cheme Gmbh, Stenheim, Germany. Atovaquone was dissolved in dimethylsulfoxide (DMSO) to concentrations of 28.8 and 6 mg/ml stock solutions. Pyrimethamine stock solution was in 1% glacial acetic acid (3 mg/ml). All solutions were stored at −20 °C until used.

### Monitoring of infection

Parasites were inoculated intraperitoneally into 10–12 weeks old BALB/c mice (approx. 1 × 10^6^ parasitized red blood cells). Peripheral blood smears were prepared once every day from tail vein bleeds. The thin films were fixed in methanol and stained with Giemsa 10%. Parasitaemia level was determined under light microscopy for at least 5000 erythrocytes.

### ED_50_ determination

Effective dose test was carried out by daily treatment of infected mice with varying doses of atovaquone or pyrimethamine. Three mice were used per group of nine logarithmically increasing doses. Growth of parasites were determined daily by monitoring parasitaemia levels. ED_50_ were calculated from the growth rates employing the Regression Wizard of SigmaPlot program.

### In vivo selection of *Plasmodium yoelii* resistant clones by repeated interrupted treatment (RIT) procedure

Drug was given by intraperitoneal injection daily. The atovaquone doses used ranged from 0.01 to 1.44 mg kg^−1^ BW as described, while a single dose of 0.15 mg kg^−1^ BW was used for pyrimethamine. Treatment was started at parasitaemia level of 3–5% and interrupted when it was reduced below 0.4%, allowing parasitaemia recovery to the initial level in the absence of the drug. This incomplete treatment regime was repeated for several cycles until resistance was observed, as indicated by the continuing increase of parasitaemia in the presence of the drug.

### Determination of mutations in *cytb* and *dhfr* genes

Approximately 50 µl tail-blood of infected mouse was collected, and kept at −20 °C in 1.5 ml heparinized Eppendorf tube. Parasites DNA was isolated from saponin (Sigma-Aldrich, St. Louis, MO, USA) lysed blood by treatment with chelex-100 (Sigma-Aldrich, St. Louis, MO, USA) essentially as described by Wooden et al. [17]. DNA was used immediately for PCR process or stored at −20 °C.

Fragments of the mitochondrial *cytb* gene were amplified by PCR employing primer pairs forward-3700 5′-TGGATGGTGTTTTAGATACTGC and reverse-4615 5-GTTTGCTTGGG AGCTGTAATC, which incorporates the Qo1 and Qo2 domains. PCR reaction was carried out for 34 cycles of 15 s denaturation at 94 °C (first cycle 5 min), 15 s annealing at 55 °C and 2 min extension at 72 °C (final extension 5 min). To amplify the *dhfr* gene, primer pair forward-1 5′-GCAATTTGTGCATGTTGTAAAGTT and reverse-570 5′-AATTTACTTAA CACACCAACACCTG were employed. PCR was run for 29 cycles of denaturation at 94 °C for 30 s (first cycle 5 min), annealing at 55 °C for 30 s and extension at 70 °C for 2 min (final extension 5 min). The PCR products obtained were directly sequenced using the forward primers to generate sequences in an ABI 377 automatic sequencer. The sequences were aligned using the BioEdit program.

## Results

### Rapid development of *P. yoelii* resistance to atovaquone and pyrimethamine during repeated interrupted malaria treatment (RIT)

**Fig. 1:**
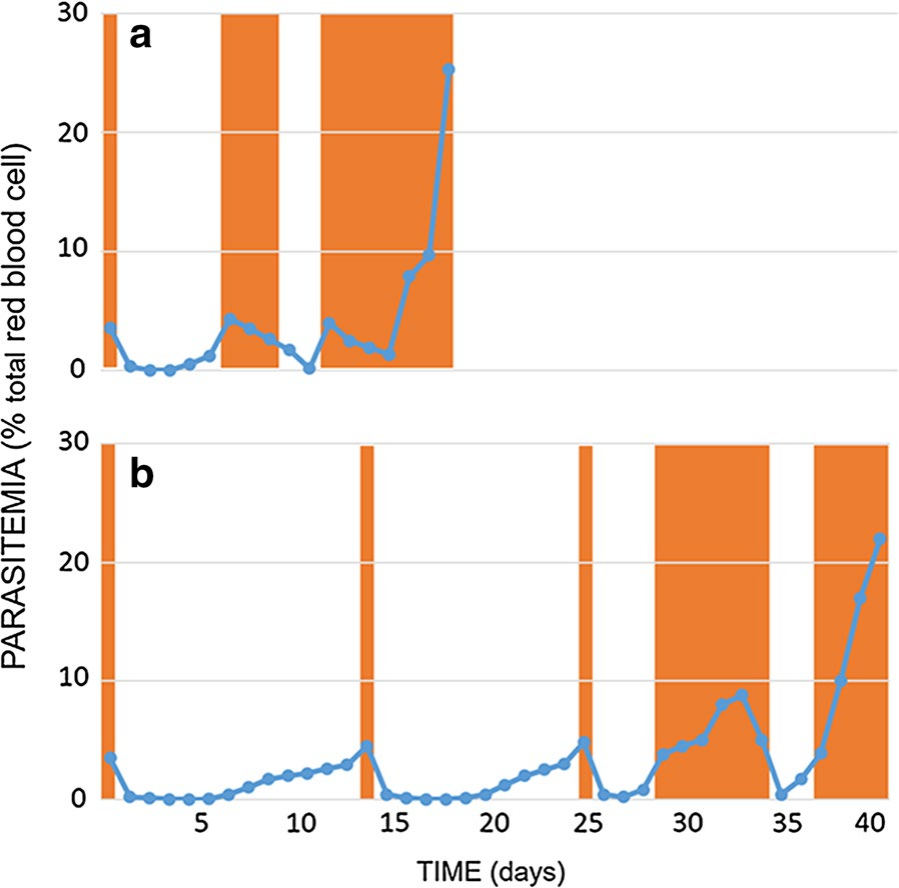
Development of resistance to atovaquone (PyASN10) and pyrimethamine (PyPSN3) following repeated interrupted treatment (RIT) of *P. yoelii* in mouse model. Shaded areas indicate the treatment periods. Stable resistant phenotype was observed in the third (**a**) and fifth (**b)** treatment cycles for atovaquone and pyrimethamine, respectively

The typical RIT experiments shown in Fig. 1, illustrating the cycles of interrupted treatment of *P. yoelii*-infected mice with atovaquone or pyrimethamine, demonstrates that stable resistant phenotype was already established in the third treatment cycle with atovaquone (Fig. 1a), compare to five treatment cycles for pyrimethamine (Fig. 1b). To establish the generality and reproducibility of the above observations, the experiments were repeated with larger numbers of mice (Table 1). The average number of treatment cycles for the development stable resistant phenotype to atovaquone was found to be 2.75 ± 1.06 (mean ± SD, n = 16), significantly lower than that to pyrimethamine (5.4 ± 0.89; n = 5; p < 0.0001).

**Table 1:** Rapid development of *P. yoelii* resistance to atovaquone and pyrimethamine

Drugs (mg kg^−1^ BW)	Isolates	Days of treatment-recovery							Cycles to resistance^a^
		1	2	3	4	5	6	7	
Atovaquone (1.44)	PyASN1	1-3	1-2	6-•					3
	PyASN2	1-5	1-5	1-3	5-•				4
	PyASN3	1-4	1-3	6-•					3
	PyASN4	1-4	1-5	3-2	4-•				4
	PyASN5	1-8	2-2	1-1	1-1	4-•			5
	PyASN6	1-2	1-5	4-•					3
	PyASN7	1-3	1-1	6-•					3
	PyASN8	1-3	1-1	6-•					3
	PyASN9	1-2	1-3	4-•					3
	PyASN10	1-5	3-2	7-•					3
Atovaquone (0.1)	PyASN11	4-6	9-•						2
Atovaquone (0.3)	PyASN12	4-3	10-•						2
	PyASN13	4-3	12-•						2
Atovaquone (0.03)	PyASN14	13-•							1
	PyASN15	14-•							1
Atovaquone (0.01)	PyASN16	12-2	8-•						2
Pyrimethamine (0.15)	PyPSN1	1-11	1-24	1-2	2-4	3-3	2-2	2-•	7
	PyPSN2	1-5	1-24	3-2	6-3	3-•			5
	PyPSN3	1-12	1-10	1-3	6-2	4-•			5
	PyPSN4	1-4	1-26	2-2	3-3	3-•			5
	PyPSN5	1-11	1-10	1-3	2-2	2-•			5

^a^One cycle consists of days of treatment (to bring parasitaemia level down to below 0.4%)—days of recovery (to bring parasitaemia level up to return to 3–5%). • = termination of treatment cycles due to the development of stable resistance (parasitaemia level >25%). In the case of PyASN14 and PyASN15, the development of resistance occurred in less than one cycle of treatment

### Mutations underlying *P. yoelii* resistance to atovaquone selected by RIT are dose dependent

Table 2 shows that almost all mutations that underlie *P. yoelii* resistance to 0.3–1.44 mg kg^−1^ BW of atovaquone, were single mutations in the Qo2 domain of the *cytb* gene (I258M, F267L/S/I, L271V, and K272R); one was found to be a double mutant of L271V and K272R. When treated with low doses of 0.01–0.03 mg kg^−1^ BW of atovaquone, the underlying resistance mutations were found to be M133I and T139S, both in the Qo1 domain. These findings demonstrated the dose dependent effect of atovaquone resistance selection in *P. yoelii*.

**Table 2:** Mutations underlie *P. yoelii* resistance to atovaquone

Doses	Isolates	Mutations	
		Qo2	Qo1
1.44	PyASN1	L271V+K272R	–
	PyASN2	L271V	–
	PyASN3	F267L	–
	PyASN4	F267S	–
	PyASN5	K272R	–
	PyASN6	K272R	–
	PyASN7	K272R	–
	PyASN8	K272R	–
	PyASN9	F267L	–
	PyASN10	K272R	–
1	PyASN11	I258M	–
0.3	PyASN12	F267I	–
	PyASN13	F267S	–
0.03	PyASN14	–	T139S
	PyASN15	–	M133I
0.01	PyASN16	–	M133I

Doses were in mg kg^−1^ BW. Qo1 and Qo2 domains were parts of Qo site of mitochondria *cytb* gene

### The dynamic of within-host resistance selection of *P. yoelii* to atovaquone during RIT

As shown in Table 1, the number of treatment cycles that lead to resistance phenotype for isolates PyASN1 to PyASN13, with mutations in the Qo2 domain of *cytb* gene, was 3.08 ± 0.86 (mean ± SD, n = 13). While those for isolates PyASN14 to PyASN16, with mutations in Qo1 domain were 1.33 ± 0.58 (n = 3). Although the selection of the resistance mutations in the Qo2 domain required more treatment cycles than in Qo1 domain (p = 0.0054), the total number of days to develop the resistance phenotype were found to be similar (16.15 ± 3.67 and 16.33 ± 4.93, respectively), as the treatment time required to lower parasitaemia level to less than 0.4%, was much longer for the low doses associated with mutations in the Qo1 (13 days) compare to only 2 days for the higher therapeutic doses of mutations in the Qo2 domains (Table 1).

### A novel mutation that underlie of *P. yoelii* resistance to pyrimethamine after RIT

The selection for pyrimethamine resistance of *P. yoelii* was not performed as extensively as atovaquone (only one dose of 0.15 mg kg^−1^ WB was used), because only one point mutation, N110S in the *dhfr* gene, has been observed in RIT of *P. berghei* [1]. Interestingly, out of five mutations that underlie resistance to pyrimethamine in *P. yoelii* (isolates PyPSN1 to PyPSN5), four were found to result in amino acid change from asparagine to cysteine (N106C), and only one (N106S) was equivalent with the N110S in *P. berghei.*

## Discussion

The findings of this study confirmed that the rapid development of resistance to atovaquone and pyrimethamine following RIT is also true for *P. yoelii*, and is not peculiar to *P. berghei.* Thus, the number of treatment cycles for the development stable resistant phenotype to atovaquone and pyrimethamine were found to be 2.75 ± 1.06 and 5.4 ± 0.89, respectively, similar to the number of cycles observed in *P. berghei* (2.47 ± 0.70 and 5.44 ± 1.46, respectively) [1]. The observation that resistance to atovaquone developed significantly faster than that for pyrimethamine is consistent with the rate of mitochondrial DNA mutation being higher than that of the nucleus [12].

Of significant was the observation that the mutations which underlie *P. yoelii* resistance to atovaquone were systematically associated with two functional domains of the cytochrome *b* protein, Qo1 and Qo2, involved in the interaction of the protein with coenzyme Q. All mutations resulting from repeated incomplete treatment with the higher therapeutic doses of 0.3–1.44 mg kg^−1^ BW were found to be in the Qo2 domain, while those resulting from repeated treatment with presumably sub-therapeutic doses of 0.01–0.03 mg kg^−1^ BW were associated with the Qo1 domain.

Despite the markedly higher sensitivity of *P. yoelii* to atovaquone (ED_50_ 0.001; compare to 0.01 for *P. berghei* [3]), most of the resistance mutations were those previously reported in *P. berghei* (M133I in Qo1 domain; L271 and K272R in Qo2 domain); the *P. yoelii* T139S mutation is at the same site as T139N mutation in *P. berghei*. Four resistance mutations at two sites in Qo2 (I258M and F267I/L/S) had not been observed in a previous study of *P. berghei*, although mutation I258M and F267I have been reported in atovaquone resistance mutants of *P. yoelii* isolated by serial technique (ST) [4]. The atovaquone resistance mutations observed following the RIT of *P. berghei* and *P. yoelii* confirmed the putative drug binding sites suggested in *Plasmodium falciparum;* the M133I, I258M, F267I/L/S and L271 mutations observed in the murine malaria parasites occurred at the putative contact residues of atovaquone binding sites (I119, F123, Y126, M133, V140, I141, L144, I258, P260, F264, F267, Y268, L271, V284, L285, and L288 [2]).

Of interest are resistance mutations at the putative contact residues L271 and its neighboring K272. In this study, they were found to be mostly single mutations K272R (five) or L271V (one), instead of as double mutations (L271V+K272R) observed following RIT of *P. berghei.* In atovaquone resistance mutants isolated by ST, mutations in the two sites are usually found to coexist with other mutations at contact residues M133 [2] or L271 [4]. This suggests that the development of high resistance to atovaquone during ST, which involves challenges to gradually increasing doses of the drug, are associated with the selection of M133I or L271V at the lower dose of atovaquone, followed by the appearance of K272R as a second mutation, conferring resistance to the higher dose of atovaquone.

The dose dependent phenomenon in the selection of resistance mutations to atovaquone in *P. yoelii* during RIT, might reflect the outcome of two different causes of malaria treatment failure in human; repeated incomplete treatment with therapeutic dose and repeated inadequate treatment associated with sub-therapeutic dose. It is thus important to confirm, and systematically investigate, that such dose dependent selection is also true for *P. berghei*. RIT models reflecting the above causes of treatment failure could be developed as useful tools to predict the potential emergence of resistance, including to newly introduced compounds, providing knowledge essential for planning malaria control and devising strategies to delay the emergence of resistance.

## Conclusions

RIT of *P. yoelii* infected mice led to rapid development of resistance to atovaquone and pyrimethamine, with dose dependent sites of mutations in the cytochrome *b* gene for atovaquone; in the Qo2 region following RIT with high therapeutic doses, in the Qo1 region with low sub-therapeutic doses. RIT models could be developed to specifically reflect two different causes of malaria treatment failure in human, as useful tools to predict the potential emergence of resistance: repeated incomplete treatment with therapeutic dose (RIcT [1]) and repeated inadequate treatment associated with sub-therapeutic dose (RIaT).

## Abbreviations

RIT: repeated interrupted treatment
RIcT: repeated incomplete treatment
RIaT: repeated inadequate treatment
*cytb*: cytochrome *b*
*dhfr*: dihydrofolate reductase
DMSO: dimethyl-sulfoxide
DNA: deoxyribonucleic acid
ST: serial technique
